# Near‐Field Photodetection in Direction Tunable Surface Plasmon Polaritons Waveguides Embedded with Graphene

**DOI:** 10.1002/advs.202302707

**Published:** 2023-09-03

**Authors:** Chia‐Hung Wu, Chih‐Jen Ku, Min‐Wen Yu, Jhen‐Hong Yang, Pei‐Yuan Wu, Chen‐Bin Huang, Tien‐Chang Lu, Jer‐Shing Huang, Satoshi Ishii, Kuo‐Ping Chen

**Affiliations:** ^1^ College of Photonics National Yang Ming Chiao Tung University 301 Gaofa 3rd Road Tainan 71150 Taiwan; ^2^ Institute of Imaging and Biomedical Photonics College of Photonics National Yang Ming Chiao Tung University 301 Gaofa 3rd Road Tainan 71150 Taiwan; ^3^ Institute of Photonics Technologies National Tsing Hua University Hsinchu 300 Taiwan; ^4^ Department of Photonics College of Electrical and Computer Engineering National Yang Ming Chiao Tung University Hsinchu 30010 Taiwan; ^5^ Leibniz Institute of Photonic Technology Albert‐Einstein Straße 9 07745 Jena Germany; ^6^ Institute of Physical Chemistry and Abbe Center of Photonics Friedrich‐Schiller‐Universität Jena Helmholtzweg 4 D‐07743 Jena Germany; ^7^ Research Center for Applied Sciences Academia Sinica 128 Academia Road, Sec. 2, Nankang District Taipei 11529 Taiwan; ^8^ Department of Electrophysics National Yang Ming Chiao Tung University No. 1001 Daxue Rd, East District Hsinchu 30010 Taiwan; ^9^ Research Center for Materials Nanoarchitectonics (MANA) National Institute for Materials Science (NIMS) 1‐1 Namiki Tsukuba Ibaraki 305‐0044 Japan

**Keywords:** graphene, photodetectors, surface plasmon polaritons, unidirectional propagation, waveguides

## Abstract

2D materials have manifested themselves as key components toward compact integrated circuits. Because of their capability to circumvent the diffraction limit, light manipulation using surface plasmon polaritons (SPPs) is highly‐valued. In this study, plasmonic photodetection using graphene as a 2D material is investigated. Non‐scattering near‐field detection of SPPs is implemented via monolayer graphene stacked under an SPP waveguide with a symmetric antenna. Energy conversion between radiation power and electrical signals is utilized for the photovoltaic and photoconductive processes of the gold‐graphene interface and biased electrodes, measuring a maximum photoresponsivity of 29.2 mA W^−1^. The generated photocurrent is altered under the polarization state of the input light, producing a 400% contrast between the maximum and minimum signals. This result is universally applicable to all on‐chip optoelectronic circuits.

## Graphical abstract

This study explores plasmonic photodetection using graphene, achieving non‐scattering near‐field detection of surface plasmon polaritons. The maximum photoresponsivity is 29.2 mA W^−1^, and the polarization state of input light produces a 400% contrast. This has potential applications in on‐chip optoelectronic circuits.

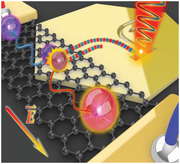

## 1 Introduction

The unique properties of light, along with rapid advancements in quantum technology and next‐generation semiconductors, has driven researchers to explore innovative approaches for light manipulation and signal modulation. Specified by the photon energy, light has properties such as the primitive and orbital forms of angular momentum.^[^ ^1^ ^]^ Due to their intriguing interactions with matter at nanoscale, the spins and orbitals of photons have gained considerable attention.^[^ ^1^, ^2^ ^]^ Optical communication technologies have been developed for global data traffic, cloud and data‐center optical interconnects, high‐performance computing, 5G and B6G, military applications, astronomy systems, and chip‐scale integrated circuits. Researchers have actively studied silicon photonics to comply with Moore's law. A system with optical interconnections between or within devices actualizes a broader bandwidth and faster data transfer.^[^ ^3^ ^]^

However, on‐chip silicon waveguides scale up to tens of micrometers, which is not sufficiently small compared to electronic components. Plasmonics uses the capability of metals to trap electromagnetic fields at deep‐subwavelength scale, allowing considerable scaling down of devices. This reduction in size aids in welding optical and electronic elements of the same size, introducing optoelectronic integrated circuits. SPP is the delocalized oscillation of electrons at the metal–dielectric interface when light couples with plasmon frequency under required conditions.^[^ ^4^ ^]^ Light propagating in this form has significant field confinement. Owing to their ability to circumvent the diffraction limit, plasmonic nanostructures have attracted much interest, and a wide variety of designs have been reported in recent years. Plasmonic devices such as nanowaveguides,^[^ ^5^, ^6^, ^7^ ^]^ plasmonic nanolasers,^[^ ^6^, ^8^, ^9^ ^]^ circulators,^[^ ^10^ ^]^ and nanoantennas have been developed to advance further plasmonic circuitry technology.^[^ ^11^, ^12^, ^13^, ^14^ ^]^ The conventional approach to detect SPPs is to break the homogeneity of the propagation route such that the waves couple out of the surface and collect the scattered light in the far field.^[^ ^15^ ^]^ Multiple optical elements and bulky systems are required to observe the SPPs radiation. Therefore, the compact and on‐chip SPP detection system is highly demanded.

Semiconductor‐based photodetectors, including silicon,^[^ ^16^, ^17^ ^]^ germanium,^[^ ^2^, ^16^ ^]^ perovskite,^[^ ^18^, ^19^, ^20^ ^]^ and III‐V materials have progressed remarkably due to their capability to bridge the gap between optical and electrical signals.^[^ ^21^ ^]^ However, the conventional semiconductor photodetectors are with the thickness in µm scale. Furthermore, the band gaps of these semiconductors facilitate a narrow range of practical applications. Graphene is a one‐atom‐thick carbon sheet packed in a hexagonal lattice and is known for its excellent optical, mechanical and electrical properties.^[^ ^22^ ^]^ Because of its transparency and flexibility, it has been applied in wearable sensors.^[^ ^23^ ^]^ Graphene has a gapless band structure that allows the absorption of light from 300 to 2500 nm and an accessible interband transition. The high carrier mobility in graphene guarantees an ultrafast response of these photogenerated carriers, which is promising for photodetector applications and integrated photonics.^[^ ^7^, ^24^, ^25^, ^26^, ^27^, ^28^, ^29^ ^]^

This study investigates a direction‐tunable SPP graphene photodetector. Pioneering the use of graphene for detecting polarization steered SPP propagation integrated with a subwavelength nanoantenna and plasmonic waveguide, creates a new paradigm in reconfigurable plasmonic photodetection systems. Previous studies have demonstrated that asymmetric nanostructures can realize directional launching of SPPs.^[^ ^15^, ^30^ ^]^ However, this design has limitations in its application spectrum because the propagation direction is fixed upon fabrication. Alternatively, SPP steering can be realized using symmetric nanostructures under different excitation conditions.^[^ ^1^, ^2^, ^10^, ^14^ ^]^ This approach enables multiport signal transfer and is presumably applicable to all plasmonic‐integrated circuits. The proposed SPP waveguide was fabricated on a single‐crystalline gold flake to minimize unnecessary propagation loss due to the inhomogeneous lattice orientation and surface roughness of the evaporated films. A graphene sheet was placed under the waveguide to collect excited SPPs and convert them into electrical signals. The mechanism of converting photoenergy into electrical signals in graphene can be broadly classified into two categories, namely, carrier generation and thermal effects. The former involves carrier generation, including the photovoltaic effect (PV),^[^ ^31^, ^32^, ^33^, ^34^ ^]^ photoconductive effect (PC)^[^ ^31^, ^35^ ^]^ and photogating effect (PG).^[^ ^36^, ^37^ ^]^ The latter include the photothermoelectric effect (PTE)^[^ ^38^, ^39^ ^]^ and bolometric effect (BOL).^[^ ^40^, ^41^ ^]^ The PV effect entails the generation of photo‐induced carriers in the graphene channel. Once photons are absorbed by the graphene sheet, electron‐hole pairs (EHPs) separate to form free carriers that diffuse by introducing a usable electromotive force (EMF), usually proportional to the irradiance power. The diffusion force originates from the divergence of the material band structure, also known as the p‐n junction in semiconductors. Graphene can be modified as p‐ or n‐type by applying a gate voltage or placing materials with different work functions underneath the sheet. However, the recombination length based on the internal electric field of the free carriers barely exceeds 200 nm.^[^ ^34^ ^]^ Therefore, incorporating an external electric field could facilitate carrier collection efficiency at the source‐drain terminals. The photoconductive effect occurs when the radiation energy is adequately high to excite the electrons in graphene from the valence band to the conduction band. This interband transition enables the free movement of electrons, thereby enhancing the conductivity of the sheet.^[^ ^35^ ^]^ The photogating process occurs when a particular type of charged carriers are trapped in graphene, providing an ultrahigh gain in responsivity. This trap occurs in gated field effect transistors (FETs) and graphene heterostructures.^[^ ^36^, ^37^ ^]^ The photo‐thermoelectric process depends on the temperature gradient of the graphene channel induced by light illumination. The majority type of carriers (n‐type or p‐type) are driven from the hot side toward the cold side of the channel, and the net carrier migration leads to a detectable photocurrent response.^[^ ^39^ ^]^ In highly doped graphene photodetectors, the bolometric process dominates the detection response. The resistivity of the graphene channel is modulated by the thermal disruption of local carriers caused by the incident light.^[^ ^42^ ^]^ This retards the charged‐carrier mobility characterized by the bolometric coefficient $\beta =\frac{d\sigma }{dT}$, contributing to a bolometric photocurrent response that is opposite in polarity to the given bias current (I_DS_).^+−^ Regarding multiple mechanisms, our proposed polarization‐dependent photodetector was based on a fusion of the PV and PC effects, which introduced a device with low power consumption and fast response. The PTE effect can be neglected owing to the large channel length and outstanding thermal conductivity of graphene. The conditions for the BOL effect are investigated further in this manuscript. It is difficult to decouple these effects; however, with careful design, engineering these mechanisms constitutes a crucial step in the optoelectronic industry.

## 2 Results and Discussions

### 2.1 Graphene Photodetector Device

**Figure 1:**
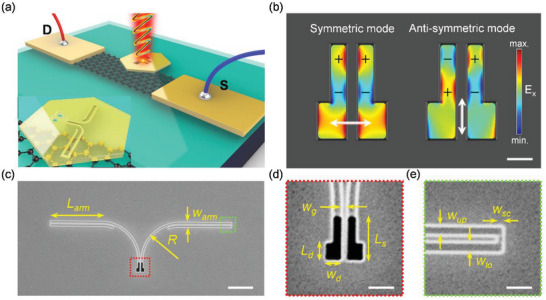
a) A schematic of SPP waveguide graphene photodetector. Inset: Transparent illustration of the patterned gold flake. Note that the antennas were not in contact with the graphene boundary to refrain direct illumination. b) Simulated electric field profiles of the designed dimer antenna showing the symmetric and anti‐symmetric modes, excited by the 0° and 90° polarizations respectively; the scale bar is 0.2 µm. c) The SEM image of the fabricated dual channel SPP waveguide; the scale bar is 2 µm. d) Magnified image of the dimer antenna to aid the eyes; the scale bar is 0.5 µm. e) Magnified image of the waveguide channel to aid the eyes; the scale bar is 0.5 µm.

A schematic diagram of the proposed photodetector (Device A) is shown in **Figure** **1**a. 300 nm of SiO_2_ was deposited on a silicon substrate. To form the graphene channel, graphene was prepared by chemical vapor deposition (CVD), transferred to the substrate, and etched by oxygen plasma for 40 s. A shadow mask was deposited by thermal evaporation on 200 nm gold electrodes. Finally, a single‐crystal gold flake was grown on a silicon substrate and patterned via focused ion beam (FIB). The flake with the plasmonic structure was flipped upside down and partially transferred onto the channel, as shown in the inset of Figure 1a. (Refer to the Methods section for a clearer step‐by‐step explanation of the fabrication process). The plasmonic waveguide contains an etched‐through antenna and groove waveguide, which avoid direct laser illumination on graphene and may generate unwanted signals larger than the SPP's contributed photocurrents.

The dimer antenna shown in Figure 1b generates SPPs that are altered by the change in incident polarization. With incident polarization states of 0° and 90° (depicted by the arrows), the dimer antenna generated symmetric and anti‐symmetric modes, respectively, as shown in Figure 1b. The dimer antenna demonstrated an identical charge arrangement when subjected to an excitation polarization of 0°, whereas the resulting charge polarity was distributed oppositely at an incident angle of 90°. Direction‐tunable SPPs based on the modes mentioned above^[^ ^10^ ^]^ were engineered through superposition.^[^ ^14^ ^]^ In this work, a 650 nm continuous wave (CW) laser was used because of its accessibility.

The main purpose of this study was to demonstrate the near‐field detection of SPPs through graphene. Therefore, the plasmonic structure could also be replaced by other designs with directional launching capability. The directional launching of SPPs has been reported and adequately studied.^[^ ^10^, ^15^, ^43^, ^44^ ^]^ The parameters of this plasmonic waveguide designated by finite‐difference time‐domain (FDTD) simulation are briefly introduced in Figure 1c. L_arm_, the length of the waveguide arm, was set to 4 µm. It is not a deciding factor for the wavelength or propagation mode but essential for keeping the confined electric fields in plane with the graphene sheet and preventing scattering loss. With the optimal design of the dimer antenna (*L*_s_ = 720 nm, *L*_d_ = 290 nm, and *W*_d_ = 240 nm), SPPs can be launched at the target wavelength effectively. The upper, lower, and grooves (*W*_up_ = 135 nm, *W*_lo_ = 145 nm, and *W*_sc_ = 80 nm) of the waveguide preserved the targeted wavelengths and granted propagation in the waveguide. The waveguide radius was optimized to R = 3 µm by FDTD simulation, which was mainly determined by the propagation length and bending loss of the SPP wave. The gap (*W*_gap_ = 110 nm) between the two SPP channels provides the spatial phase distribution required to interfere with the field profiles generated by the dimer antennas.

### 2.2 SPP Waveguide Characterization

**Figure 2:**
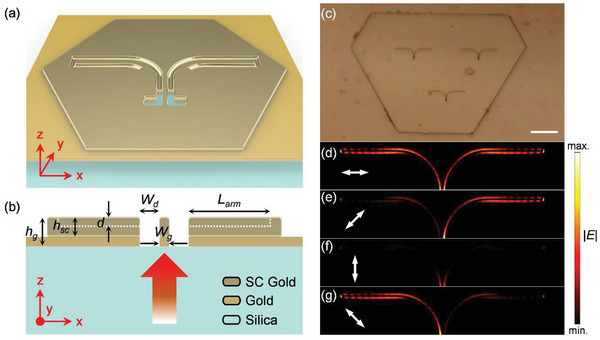
a) Schematic of the optical sample (device B). b) Cross section of the optical sample, the arrow gradient colored represents the incident beam. c) Optical microscope image obtained with the camera; the scale bar is 10 µm. The simulated electric field profiles under d) 0°, e) 45°, f) 90° and g) −45° excitations, respectively.

Before fabricating the proposed photodetector, its ability to steer SPPs with linearly polarized light was tested. Device B was fabricated using the designed SPP waveguide structure shown in **Figure** **2**a,c. To minimize propagation loss in the waveguide, a single‐crystalline gold flake with a thickness of 200 nm (*h*_sc_) was grown and transferred via poly (methyl methacrylate) (PMMA) onto a 100‐nm gold film evaporated on a glass substrate.^[^ ^45^ ^]^ The gold stack with a total thickness of 300 nm was patterned with the designed plasmonic waveguide using FIB. The waveguide groove depth (d = 120 nm) was designated by simulation, and further analysis of additional etching depths is provided in the Figure S1 (Supporting Information). Gaussian beams of 650 nm wavelength with different polarization states (0°, 45°, 90°, and – 45°) were illuminated from the backside of the sample as illustrated in Figure 2b, and the simulated electric field profile is shown in Figure 2d–g. (Detailed information on the simulation environment is provided in Figure S2a–d (Supporting Information). From the results, it is clear that the SPPs were steered due to the superposition of the modes described in Figure 1b, as all the linear polarizations could be disassembled into different weights of the horizontal and vertical vectors.

**Figure 3:**
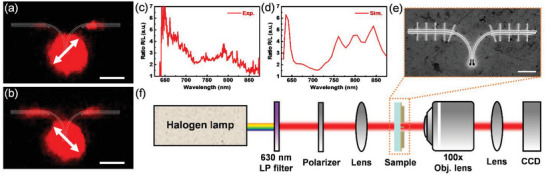
Experimental results of the SPP waveguide. Optical image of the far field measurement under a) 45° and b) −45° excitations, respectively. The scale bar is 3 µm. c) and d) are the experimental and simulated extinction ratio of scattered SPPs signals. e) SEM image of the fabricated SPP waveguide on the optical device. The scale bar is 0.2 µm. f) Schematic of the optical measurement setup.

**Figure** **3**f shows the optical measurement system configuration. A commercial halogen lamp with a 630 nm long pass filter was used as the light source. The light then passes through a polarizer to control the polarization and focuses on a higher power density with a condenser lens. The scattering of the excitations was collected using an objective lens, followed by the optical elements inside a commercial microscope, and finally, the camera and spectrometer. The experimental results of steering SPPs are shown in Figure 3a,b. Figure 3a shows the steering and scattering images when the input polarization is set to 45°. The contours in the figure have been added to aid the eyes. The experimental data are in good agreement with the near‐field profile and far‐field projection FDTD simulations shown in Figure 2e and Figure S2e (Supporting Information). The SPPs were guided toward the right channel, followed by scattering, to free space as the surface waves collided with the vertical scattering grooves, as shown in Figure 3e. Note that the scattering grooves were added only for the optical setup (Device B) and excluded from the electrical setup (Device A) to avoid scattering loss and enhance the in‐plane field absorption of graphene. With the input set to – 45°, the SPPs can be launched into the left waveguide, as shown in Figure 3b. The generated electric field confinement must be studied to analyze the largest possible photocurrent contrast. An easy way to obtain preliminary results is to calculate the extinction ratio between the energies confined in the channels of the proposed waveguide. The spectra of the right (*SC_R_*) and left (*SC_L_*) scattering ports were obtained under an excitation of 45° using a spectrometer. Figure 3c,d show the experimental and simulated extinction ratio (ER) at an excitation of 45°, where ER  =  *SC_R_*/*SC_L_*.

### 2.3 Photocurrent Alternation and Mechanisms

**Figure 4:**
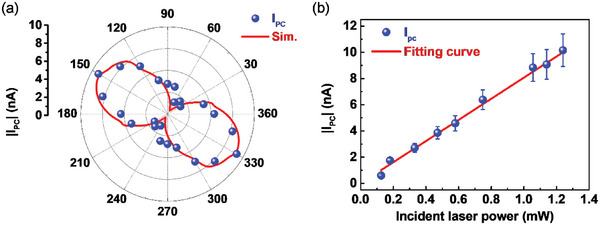
Photocurrent response and simulated SPP flux under a) full‐azimuthal polarization excitation and b) different incident laser powers of the SPP waveguide photodetector.

After the proposed plasmonic waveguide exhibited positive results in the optical far‐field characterization, a near‐field photodetector device was fabricated. The result in **Figure** **4**a shows the photocurrent alternation under different excitation polarizations, indicating promising properties for integration in integrated circuits. The simulation settings and field profiles are provided in Figure S3 (Supporting Information). The excitation instrument was a commercial 650 nm CW laser with its power set to 1.15 mW. When the polarization angle was set to 150°, the photocurrent exhibited the largest response of ≈I_PC_ = 9 nA. SPPs under this scenario were launched toward and confined in the waveguide, where the graphene channel was positioned. The local EHPs in the graphene channel is separated owing to the thermal effects caused by the SPPs in the waveguide. At an excitation angle of 60°, the SPPs would propagate along the opposite channel lacking graphene; therefore, it could be inferred that small or no photocurrent could be generated. As illustrated in Figure 4b, there is a linear relationship between the photocurrent response and the incident beam, indicating that the device operates by employing photovoltaic (PV) and photoconductive (PC) effects.^[^ ^46^ ^]^ The excitation energies are proportional to the number of photons responsible for the SPP generation intensity. In contrast, SPPs generate hot carriers that excite free electrons and holes driven by externally applied electric fields (V_DS_ = −0.3 V), thereby contributing to the photocurrent.

**Figure 5:**
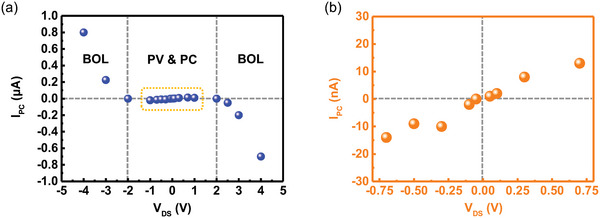
a) Bias‐dependent photocurrent response of the photodetector, presenting the conversion between operating effects. For clear observations of photocurrent response in the PV & PC region, b) is added to aid the eyes.

To further study the effects of photocurrent in graphene, a bias‐dependent experiment was conducted. The measured photocurrent exhibited a polarity equivalent to the externally applied current when |V_DS_| < 2 V. In **Figure** **5**a, the photocurrent response is zero at bias currents ‐2 and 2 V, indicating a switch in the dominant photocurrent effects. Note that the PV, PC, BOL, and PTE effects may occur simultaneously. However, by carefully designing the photodetector setup, it is possible to engineer the dominating effect. Interestingly, the photocurrent response with polarization‐dependent properties, as depicted in Figure 4a, emerged only in the PV and PC regions. The photocurrent response becomes independent of the polarization alternation in the BOL region, as shown in Figure S4 (Supporting Information). To the best of our knowledge, few studies have investigated this issue. In this state, the photocurrent obtained is due to the heat disturbance introduced by the incident beam throughout the graphene channel. The carrier mobility was suppressed, indicating an increase in channel resistance. Therefore, at high bias voltages, the measured bolometric effect was pronounced, overwhelming the other graphene photocurrent effects. As the alternation of the incident beam polarization does not affect the power, the introduced heat disturbance was the same. Because the contributing photocurrent scales of BOL and PV are significantly different, the polarization‐dependent photocurrent response feature is deprived of the device under the abovementioned conditions.

## 3 Conclusion

Optically integrated circuits have been developed to meet the ever‐growing demands of global communication technology. Photodetectors are the solutions to optical and electrical signal conversion. However, owing to size incompatibility, on‐chip integration remains an issue for conventional photodetectors. Miniaturized photodetectors have attracted significant interest in recent years.

A polarization‐dependent SPP near‐field photodetector based on graphene was developed in this work. By illuminating the backside of the groove waveguide, the antenna served as a nanoscale light source, exciting waves that propagated through the desired channel. Subsequently, the SPP waves are absorbed directly and converted into electrical signals in the near field, offering a promising near‐optical field detector based on graphene. Different photocurrents were observed under alternating incident polarization. The responsivities of the device under 150° and 60° excitation were 29.2 and 6.2 mA W^−1^, respectively, introducing a 400% electrical signal contrast for further applications in integrated circuits. In addition, the photocurrent generation mechanism of graphene in the device was investigated. The device was subjected to different V_DS_ values to measure the PV, PC, and BOL effects of graphene. The different effects of photocurrent generation can be employed under careful design and particular conditions to accommodate the vast applications of the optoelectronic device industry. For future improvement of the device performance, the integrated transition‐metal dichalcogenides (TMDCs) 2D heterostructures,^[^ ^47^ ^]^ hybrid plasmonic waveguides (HPWGs),^[^ ^48^, ^49^, ^50^ ^]^ and decreasing the drain to source channel length could be considered. Near‐field SPP detection, introduced by the fusion of photovoltaic and photoconductive effects, saves energy and avoids complicated and expensive fabrication procedures. The proposed device can be used in nanolaser‐integrated plasmonic circuits for ultracompact nanophotonic communication.

## 4 Experimental Section

### Device Fabrication

The device was fabricated by depositing a 300 mm insulating layer of silicon dioxide on a silicon substrate using an electron beam evaporation system (ULVAC VT1‐10CE). The insulating layer restricted any contact between the electrode and silicon layer. Therefore, the photocurrent contributed by silicon was not detected by the source meter (B2901A). Graphene was transferred onto the sample using the wet transfer method, followed by oxygen plasma etching^[^ ^51^ ^]^ using a reactive ion etching system to obtain a channel with uniform edges. Subsequently, the designed plasmonic waveguide was milled using an FIB on a single‐crystal gold flake grown on a silicon substrate using a wet chemical synthesis method.^[^ ^45^ ^]^ As structural deformities may affect SPP propagation in a lossy manner, the FIB working current was set to a low value (7 pA) to achieve effective results. To realize different milling depths in the waveguide and antenna, each was patterned separately using the bitmap function of the FIB (FEI Helios NanoLab G3 CX, NCKU, Taiwan). The structured flake was transferred upside down and aligned at the desired location.

### Graphene Wet Transfer

A monolayer graphene sheet grown on a copper foil by the chemical vapor deposition (CVD) method was transferred using the wet chemical technique. Graphene was protected by spin coating a thin layer of photoresist (PMMA‐A4). The copper foil was removed by placing the stack in a Fe(NO_3_)_3_ solution (33 wt.%) at room temperature (23–26°C) for ≈12 h. Consequently, the copper foil was fully etched away, leaving a photoresist/graphene stack. The stack was cleaned for 1 h using deionized (DI) water to remove etchant residue. To enhance surface energy, the target substrate was treated with UV‐ozone before the transfer, providing a location adjustable transfer process without damaging the graphene. The stack with the substrate was left in an oven at room temperature to complete dehydration, then immersed in acetone to remove the photoresist and subsequently washed in isopropanol (IPA).

### Upside‐Down Transfer of Patterned Single Crystalline Gold Flake

Few applications require the upside‐down transfer of nanostructures. In the proposed device, this technique plays a crucial role in creating a light source at the nanoscale and precludes direct illumination of the graphene sheet. First, the waveguide was patterned on a single crystalline gold flake, then 2 µL of photoresist was applied on the target flake with a pipette and left to dry on a hot plate for 2 min at 150°C. The resist/gold stack was peeled from the native substrate with tweezers, aided by a few drops of DI water on the edge. The stack was placed on a thin PDMS stamp and immersed in acetone for 2 h to reveal the gold flakes. The PDMS, along with the flake, was mounted onto a micromanipulator and aligned with the device using a microscope. The device and stamp stack were heated to 120°C to provide better contact energy between the flakes and device. Finally, the PDMS stamp was slowly peeled off from the device, leaving the patterned flake at the desired location. For clear schematics, please refer to Figure S7 (Supporting Information).

## Conflict of Interest

The authors declare no conflict of interest.

## Author Contributions

C.‐H. W., C.‐J. K., and M.‐W. Y. performed sample fabrication, simulation, and optical characterization. J.‐H. Y., P.‐Y. W., C.‐B. H., T.‐C. L., J.‐S. H., S. I. and K.‐P. C. helped analyze the experimental data. C.‐H. W., M.‐W. Y. and K.‐P.C. wrote the manuscript. All authors discussed the results and commented on the manuscript.

## Supporting information

Supporting Information

## Data Availability

The data that support the findings of this study are available from the corresponding author upon reasonable request.
