# Supplementary material for: Near‐Field Photodetection in Direction Tunable Surface Plasmon Polaritons Waveguides Embedded with Graphene
Source: Adv Sci (Weinh). 2023 Sep 3;10(30):2302707. doi: 10.1002/advs.202302707 (PMC10602515; doi:10.1002/advs.202302707)
Supplement: Supplementary file 1 — Supporting Information [file ADVS-10-2302707-s001.pdf]

ADVANCED SCIENCE
Open Access

Supporting Information

for *Adv. Sci.*, DOI 10.1002/advs.202302707

Near-Field Photodetection in Direction Tunable Surface Plasmon Polaritons Waveguides Embedded with Graphene

*Chia-Hung Wu, Chih-Jen Ku, Min-Wen Yu, Jhen-Hong Yang, Pei-Yuan Wu, Chen-Bin Huang, Tien-Chang Lu, Jer-Shing Huang, Satoshi Ishii and Kuo-Ping Chen**

Supporting Information

Near-field Photodetection in Direction Tunable Surface Plasmon Polaritons Waveguides Embedded with Graphene

Chia-Hung Wu[1], *Chih-Jen Ku*[2], *Min-Wen Yu*[1], *Jhen-Hong Yang*[1], *Pei-Yuan Wu*[3], *Chen-Bin Huang*[3], *Tien-Chang Lu*[4], *Jer-Shing Huang*[5,6,7,8], *Satoshi Ishii*[9], and *Kuo-Ping Chen*[2,3,*]

[1] College of Photonics, National Yang Ming Chiao Tung University, 301 Gaofa 3rd Road, Tainan 71150, Taiwan

[2] Institute of Imaging and Biomedical Photonics, College of Photonics, National Yang Ming Chiao Tung University, 301 Gaofa 3rd Road, Tainan 71150, Taiwan

[3] Institute of Photonics Technologies, National Tsing Hua University, Hsinchu 300, Taiwan

[4] Department of Photonics, College of Electrical and Computer Engineering, National Yang Ming Chiao Tung University, Hsinchu 30010, Taiwan

[5] Leibniz Institute of Photonic Technology, Albert-Einstein Straße 9, 07745 Jena, Germany

[6] Institute of Physical Chemistry and Abbe Center of Photonics, Friedrich-Schiller-Universität Jena, Helmholtzweg 4, D-07743 Jena, Germany

[7] Research Center for Applied Sciences, Academia Sinica, 128 Academia Road, Sec. 2, Nankang District, Taipei 11529, Taiwan

[8] Department of Electrophysics, National Yang Ming Chiao Tung University, No. 1001 Daxue Rd, East District, Hsinchu 30010, Taiwan

[9] International Center for Materials Nanoarchitectonics (MANA), National Institute for Materials Science (NIMS), 1-1 Namiki, Tsukuba, Ibaraki 305-0044, Japan

*kpchen@ee.nthu.edu.tw
KEYWORDS: Surface Plasmon Polariton, Graphene, unidirectional propagation

Simulation:

In this section, we will briefly introduce the environments of the simulation and some of the results, note that in all models, the light source application was 650 nm Gaussian Beam. Since gold grown by the chemical synthesis method has no fixed form, the thickness may alter under numerous conditions, which may somehow influence the SPPs generation. To cancel the concern, we conducted a parameter sweep simulation for thickness of gold as shown in Fig. S1(a). The monitors were transmission monitors located 1μm above the designed nanostructure, collecting SPPs scattering off the right (R) and left (L) channels. The simulation result shows that with different gold flake thickness the scattering intensity changes are neglectible. In Fig. S1(b), the waveguide depth was investigated for optimization and was designated to 90 nm according to the simulated results.

Fig. S2(a~d) shows the simulation model for device B. The orange box indicates the PML boundaries of the simulaiton region, whereas the yellow dashed box is the farfield projecting monitor with the result shown in Fig. S2(e). The excitation was a 45° polarized gaussian beam at 650 nm. According to the result, it was clear and shows good agreement with the experimental result in Fig. 3(a), radiation from SPPs could be observed in the farfield above the right channel of the structure.

However, farfield simulation was unnecessary and impractical for device A because the pattern was turned upside down facing the silicon substrate, and graphene converts the optical power to electronical signals precluding radiation loss. Fig. S3 (a) and (b) shows the model, the monitor was placed in the location of the graphene to calculate the field generated. Fig. S3(c~f) shows the field confinement conditions, which aids to expect the measured photocurrent.

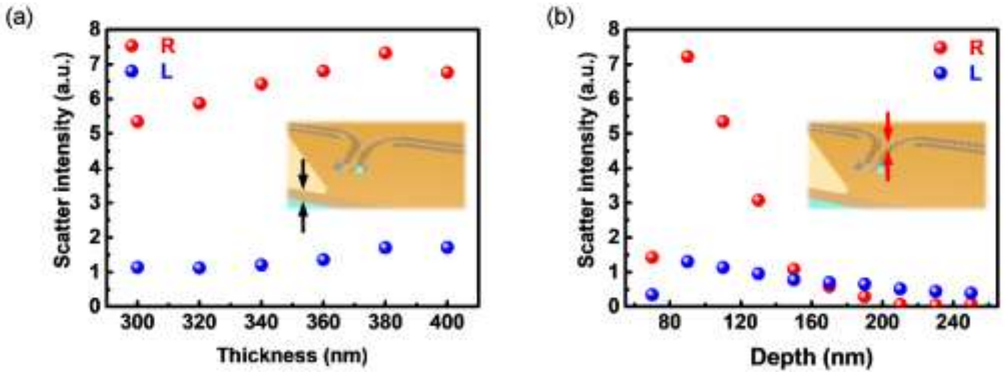

Figure S1. Simulated data of SPPs scattering intensity obtained in the far field under (a) different gold flake thickness and (b) waveguide depth for device B. The arrows in (a) and (b) were added to aid the eyes, indicating the variable in this model.

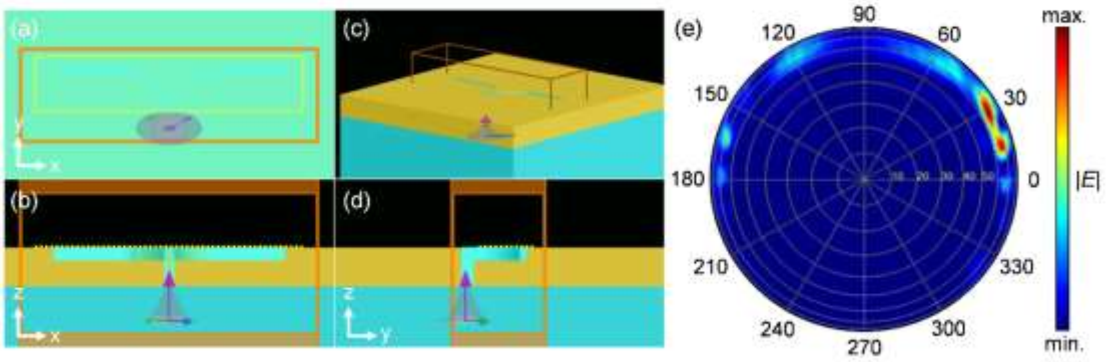

Figure S2. Simulation environment and far field profile for device B. (a) Top view, (b) front view, (c) prespective view and (d) side view. For (a ~ d), the orange box indicates the simulation region, the yellow dashed box and line labling the position of monitor receiving the far field profile in (e). The purple and green arrow in (a) shows the electric and magnetic field oscillation direction of excitation respectively.

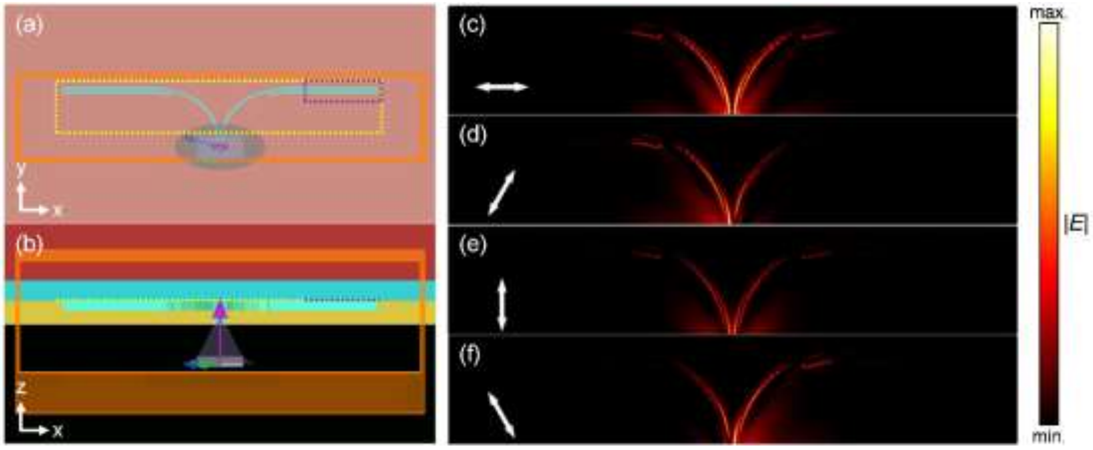

Figure S3. Simulation environment and near field profile for device A. (a) Top view, (b) front view. The orange, yellow dashed and purple dashed boxes indicates the simulation region, near field profile monitor for (c ~ f) and SPPs intensity alternation with different polarizations excitation in the manuscript Fig. 4(a) respectively. Electric field profiles of (c) 0°, (d) 60°, (e) 90° and (f) 150°.

Measurements:

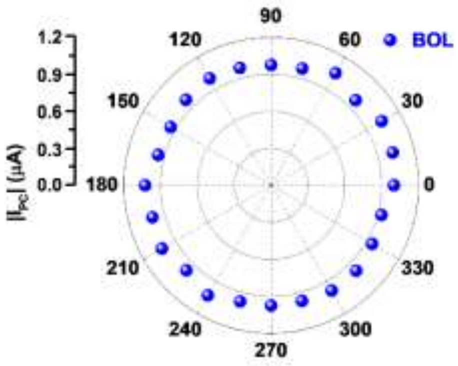

Figure S4. Photocurrent measurement of device A under V_{DS} = - 4 V.

Control Group: Device C

In order to confirm that the observed polarization-dependent results presented in Figure 4(a) of the main text originate from the proposed plasmonic waveguide. We conducted an experimental investigation involving the fabrication of a comparable device, denoted as device C. This device was constructed with the same configuration as device A, with the exception of an unpatterned gold flake, as illustrated in Figure S5(a). The absence of a structured surface precludes directional launching of SPPs, which is essential for generating a polarization dependent photocurrent. As shown in Figure S5(c) and (d), the photocurrent remains unaffected by alterations in the incident polarization in the absence of the structured surface, thus verifying the results observed in Figure 4(a) are indeed a result of the proposed plasmonic waveguide.

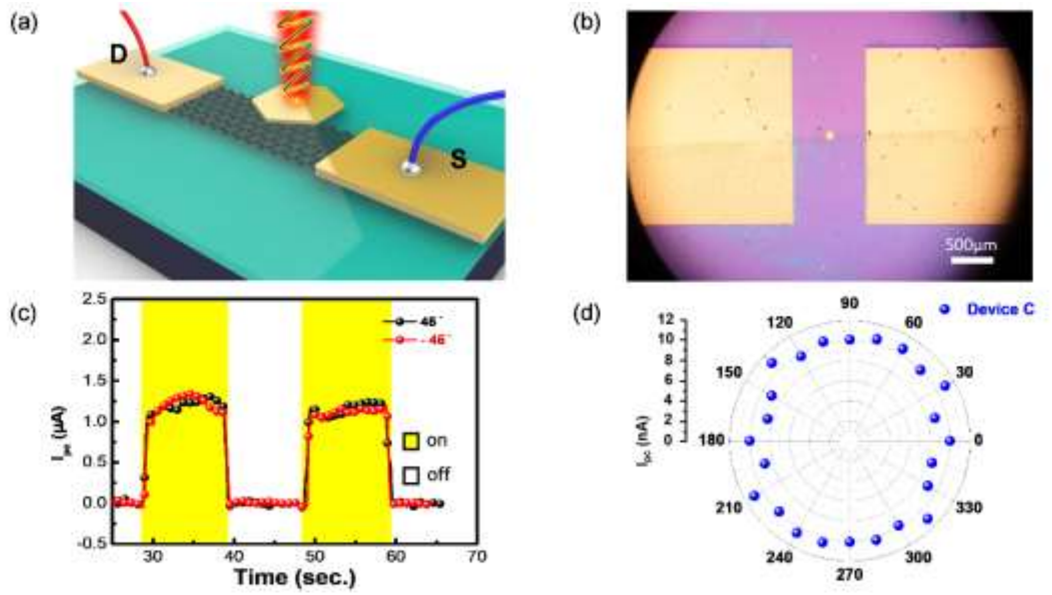

Figure S5. (a) Schematic of device C. (b) Optical microscope image of device C obtained with the camera. (c) Measuered 45° and - 45° excitation photocurrent response with V_{DS} = - 6 V, the yellow region refers to the illunation duration. (d) Measured polarization dependent photocurrent response with V_{DS} = - 0.3 V.

Detectivity:

$$D = \sqrt{\frac{A}{2}} \cdot R \cdot \frac{1}{\sqrt{e \cdot I_{dark}}} \text{ (Jones)}$$

$$D = \sqrt{\frac{\pi(20\cdot10^{-4})^2 cm^2}{2}} \cdot 29.2mA/W \cdot \frac{1}{\sqrt{1.6\cdot10^{-19}\cdot 43.4\mu A}} \text{ (Jones)}$$

$$D = 2.8 \cdot 10^{10} \text{(Jones)}$$

Abbreviations in the function are listed as follows:

D: detectivity, A: area of illumination, R: responsivity, I_{dark}: dark current.

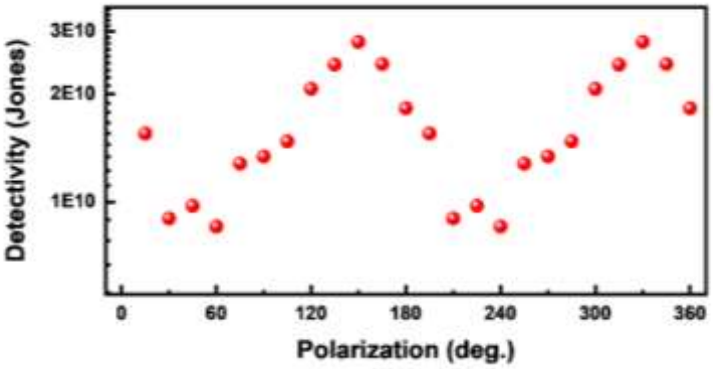

Figure S6. Polarization dependent detectivity plot of device A.

Inverted transfer method:

Normally, nanostructures based on top down process technology produces structures facing upwards. To meet our experimental requirements, an interesting transfer method was demonstrated to refrain direct illumination of graphene.

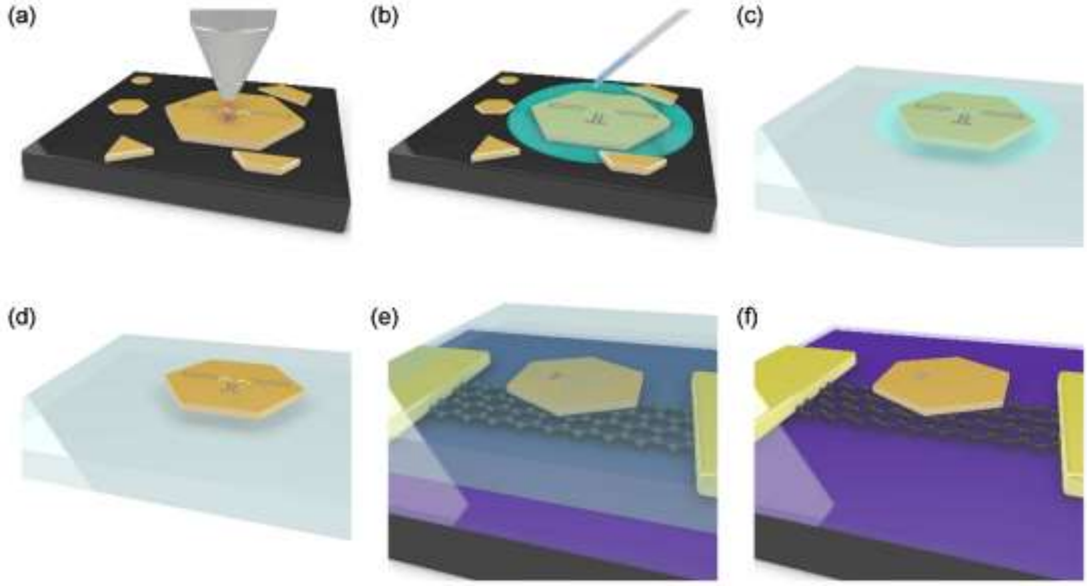

Figure S7. (a) The proposed waveguide structure was processed via focused ion beam (FIB). Then we apply (b) 2μL of photoresist (PMMA A4) on the patterned flake and dehydrated on a hot plate. (c) Gold flake/resist stack peeled off from native substrate and placed on a PDMS stamp. (d) The resist was removed via immersion in acetone. (e) PDMS stamp was flipped over and mounted on a micromanipulator and aligned with the device through a microscope. The stack was heated on a hot plate at 120°C, then (f) the stamp was peeled off slowly.

Characterization of Graphene:

The transfer method was provided in the main text of the manuscript. However, with the wet transfer method of graphene, mechanical defects like wrinkles and folds may occur. To understand the stability of our device fabricaiton, raman spectrum was measured before (as-grown on copper foil) and after (Si / SiO_2 substrate) transfer of the graphene sheet. Note that 2D and G peak ratio may alter due to measured position difference.

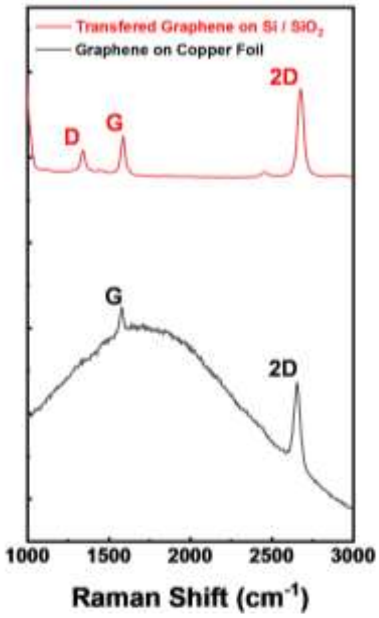

Figure S8. Raman spectrum of CVD grown graphene before and after transfer used in this device.

Table S1. Comparison of Graphene Based Plasmonic Photodetectors:

Material	Wavelength	Maximum Responsivity	Detector Type	Channel Length	Description
G./Pd/Ti/Au/SiO_2/Si	1.55 µm	6.1mA/W	PV	1 µm	P-N Junction [1]
G./Au/SiO_2/Si	Visible ~ Mid Infrared	0.26 A/W	PTE, PG	~40 µm	LSPR enhanced [2]
G./Au/Ti/SiO_2/Si	785 nm	13mA/W	PV, PTE	~50 µm	LSPR enhanced [3]
G./Au/Ti/SiO_2/Si	Visible	10 mA/W	PV	N/A	P-N Junction, LSPR enhanced [4]
G./Au/Ti/SiO_2/p-Si	Visible	24 mV/W	PV, PTE	30 µm	LSPR enhanced [5]
G./Au/Ti/SiO_2/Si	Visible	6.1 mA/W	PV, PTE	~ 8 µm	LSPR enhanced [6]
G./Au/Ni/Cr/Al_2O_3/SiN/SiO_2	1.50 ~ 1.58 µm	12.2 V/W	PTE	~4 µm	HPWG integrated [7]
G./Au/Ti/Al/Al_2O_3/SiO_2/Si	> 1.55 µm	0.4 A/W	PTE, PC, BOL	~2.8 µm	HPWG integrated [8]
G./Au/Ti/Pd/SiO_2/Si	1.54 µm	0.36 A/W	PV	120 nm	Si WG coupled SPP [9]
G./Au/Ti/SiO_2/p-Si	1.31 µm	0.67 A/W	PV, BOL	15 nm	HPWG integrated [10]
G./Au/Cr/SiO_2/Si	Visible	80 mV/W	PV, PTE	~ 2.2 µm	SPP enhanced [11]
G./Ag/Au/Ti/SiO_2/Si	633 nm	~ 45 µA/W	PV, PC	~ 4.6 µm	Plasmonic WG [12]
G./Au/Ti/SiO_2/Si	650 nm	15 mA/W	PTE, PV	~ 500 µm	Directional SPP [13]
This work	650 nm	29.2 mA/W	PC, PV	~ 1 mm	SPP Steering plasmonic WG

[1] Mueller, Thomas, Fengnian Xia, and Phaedon Avouris. "Graphene photodetectors for high-speed optical communications." *Nature photonics* 4.5 (2010): 297-301.

[2] De Nicola, Francesco, et al. "Graphene plasmonic fractal metamaterials for broadband

photodetectors." *Scientific Reports* 10.1 (2020): 6882.
[3] Fang, Zheyu, et al. "Graphene-antenna sandwich photodetector." *Nano letters* 12.7 (2012): 3808-3813.
[4] Echtermeyer, T. J., et al. "Strong plasmonic enhancement of photovoltage in graphene." *Nature communications* 2.1 (2011): 458.
[5] Fang, Jieran, et al. "Enhanced graphene photodetector with fractal metasurface." *Nano letters* 17.1 (2017): 57-62.
[6] Liu, Yuan, et al. "Plasmon resonance enhanced multicolour photodetection by graphene." *Nature communications* 2.1 (2011): 579.
[7] Muench, Jakob E., et al. "Waveguide-integrated, plasmonic enhanced graphene photodetectors." *Nano letters* 19.11 (2019): 7632-7644.
[8] Guo, Jingshu, et al. "High-performance silicon− graphene hybrid plasmonic waveguide photodetectors beyond 1.55 μm." *Light: Science & Applications* 9.1 (2020): 29.
[9] Ding, Yunhong, et al. "Ultra-compact integrated graphene plasmonic photodetector with bandwidth above 110 GHz." *Nanophotonics* 9.2 (2020): 317-325.
[10] Ma, Zhizhen, et al. "Compact graphene plasmonic slot photodetector on silicon-on-insulator with high responsivity." *ACS Photonics* 7.4 (2020): 932-940.
[11] Echtermeyer, T. J., et al. "Surface plasmon polariton graphene photodetectors." *Nano Letters* 16.1 (2016): 8-20.
[12] Ee, Ho-Seok, et al. "Long-range surface plasmon polariton detection with a graphene photodetector." *Optics letters* 43.12 (2018): 2889-2892.
[13] Wu, Chia-Hung, et al. "Nonscattering photodetection in the propagation of unidirectional surface plasmon polaritons embedded with graphene." *ACS applied materials & interfaces* 14.26 (2022): 30299-30305.
